# K_**3**_EDTA Vacuum Tubes Validation for Routine Hematological Testing

**DOI:** 10.5402/2012/875357

**Published:** 2012-07-24

**Authors:** Gabriel Lima-Oliveira, Giuseppe Lippi, Gian Luca Salvagno, Martina Montagnana, Giovanni Poli, Giovanni Pietro Solero, Geraldo Picheth, Gian Cesare Guidi

**Affiliations:** ^1^Laboratory of Clinical Biochemistry, Department of Life and Reproduction Sciences, University of Verona, 37129 Verona, Italy; ^2^Post-Graduate Program of Pharmaceutical Sciences, Department of Medical Pathology Federal University of Parana, 80210-170 Curitiba, PR, Brazil; ^3^MERCOSUL: Sector Committee of Clinical Analyses and In Vitro Diagnostics–CSM 20, 20270-902 Rio de Janeiro, RJ, Brazil; ^4^Brazilian Society of Clinical Analyses on Sao Paulo State, 02965-140 Sao Paulo, SP, Brazil; ^5^Laboratory of Clinical Chemistry and Hematology, Department of Pathology and Laboratory Medicine, Academic Hospital of Parma, 43126 Parma, Italy

## Abstract

*Background and Objective*. Some in vitro diagnostic devices (e.g, blood collection vacuum tubes and syringes for blood analyses) are not validated before the quality laboratory managers decide to start using or to change the brand. Frequently, the laboratory or hospital managers select the vacuum tubes for blood collection based on cost considerations or on relevance of a brand. The aim of this study was to validate two dry K_3_EDTA vacuum tubes of different brands for routine hematological testing. *Methods*. Blood specimens from 100 volunteers in two different K_3_EDTA vacuum tubes were collected by a single, expert phlebotomist. The routine hematological testing was done on Advia 2120i hematology system. The significance of the differences between samples was assessed by paired Student's *t*-test after checking for normality. The level of statistical significance was set at *P* < 0.05. *Results and Conclusions*. Different brand's tubes evaluated can represent a clinically relevant source of variations only on mean platelet volume (MPV) and platelet distribution width (PDW). Basically, our validation will permit the laboratory or hospital managers to select the brand's vacuum tubes validated according to him/her technical or economical reasons for routine hematological tests.

## 1. Introduction

The procedures involving phlebotomy, for example, time of tourniquet application [1–4] and blood drawing using vacuum tubes [5, 6] are poorly studied as potential sources of errors. In the daily practices of laboratory medicine, several commercial diagnostic products are involved during the testing process. All diagnostic products can be divided into two major categories: in vitro diagnostic (IVD) devices, such as laboratory instruments, reagents, assays, and blood collection tubes, and medical devices, such as specimen collection devices (needles and sets) [7]. Necessary improvements and potential sources of nonconformities, either technical or concerning the quality management system, shall be identified and all laboratory process shall be validated [8]. Some IVD devices (e.g., blood collection vacuum tubes and syringes for blood analyses) are not validated before the quality laboratory managers decide to start using or to change the brand [9]. Frequently the laboratory or hospital managers select the vacuum tubes for blood collection based on cost considerations or on relevance of a brand [10]. One important question that all quality laboratory managers need to answer is as follows: low cost is synonym of low quality or of large scale production thus implying economy? It is well known that there are several different brands of vacuum tubes for blood collection and a huge competition exists as for the producers to sell their own IVD devices. The aim of this study was to validate two dry K_3_EDTA vacuum tubes of different brands for routine hematological testing.

## 2. Material and Methods

### 2.1. Study Design

A group of 100 apparently healthy volunteers from University of Verona, Italy, consented to blood withdrawal after twelve hours fasting. This study was submitted and approved by our Internal Review Board (IRB) and all volunteers signed informed consent. 

### 2.2. Collection of Diagnostic Blood Specimens

The collection of all diagnostic blood specimens was performed by a single, expert phlebotomist, following the international standard from Clinical Laboratory Standard Institute-CLSI [11]. All volunteers were maintained seated during 15 minutes prior to phlebotomy in order to eliminate possible interferences of blood distribution due to different posture [12]. After this interval, a vein was located on forearm by a subcutaneous tissue transilluminator device (Venoscópio IV plus, Duan do Brasil, Brazil) in order to avoid the venous stasis interference [1–3], and two amounts of blood were consecutively drawn by venipuncture with 20G straight needles (Terumo Europe NV, Leuven, Belgium), directly into two different brands of vacuum tubes containing Ethylenediamine Tetraacetic Acid Tripotassium Salt (K_3_EDTA); Tube I: 4.5 mL Labor Import lot 20100505 (concentration of K_3_EDTA inside the tube is not made known by Shandong Weigao Group Medical Polymer, Weihai, China); Tube II: 1.2 mL S-Monovette lot 0092201 with 1.6 mg K_3_EDTA (Sarstedt, Nümbrecht, Germany). To eliminate any possible interferences due to either the contact phase or the tissue factor, about 2 mL blood were preliminarily collected in a tube without additive Vacuette lot A101004D (Greiner Bio-One GmbH, Kremsmünster, Austria) before the sequence above reported, and then discarded. All the samples were collected into the same type and lot of vacuum tubes.

### 2.3. Laboratory Testing

All the samples were processed for routine hematological testing immediately after collection (<15 min) on the same Advia 2120i hematology system (Siemens Healthcare Diagnostics, Deerfield, IL, USA). The parameters tested included red blood cells count (RBC), haematocrit (HCT), haemoglobin (HGB), mean red cell volume (MCV), mean red cell haemoglobin content (MCHC), red blood cell distribution width (RDW), white blood cells (WBC) count, and WBC differential, including lymphocytes (LYMPHO), monocytes (MONO), neutrophils (NEU), eosinophils (EOS), basophils (BASO) and large unstained cells (LUC), platelet count (PLT), mean platelet volume (MPV), and platelet distribution width (PDW), The instrument was calibrated against appropriate proprietary reference standard material and verified with the use of proprietary controls. A multicenter evaluation of the within-run precision of the Advia 2120 system showed coefficients of variation ranging from 1.6% to 2.3% for WBC, from 2.1% to 2.8% for platelets, from 0.6% to 0.9% for RBC, and always lower than 0.7% for hemoglobin, MCV and MCH [13].

### 2.4. Statistical Analysis

The significance of the differences between two different manufactures of K_3_EDTA vacuum tubes for collection of diagnostic blood specimens was assessed by paired Student's *t*-test after checking for normality by the D'Agostino-Pearson omnibus test [14]. As nonnormal distribution was found for MCV, MCHC, RDW, and PDW, results were assessed by Wilcoxon ranked-pairs test. The level of statistical significance was set at *P* < 0.05. Finally, the biases from Tube I and Tube II were compared with the current desirable quality specifications for bias (*B*), derived from biological variation according to the formula *B* < 0.25(CV_*w*_^2^ + CV_*g*_^2^)^1/2^ where CV_*w*_ and CV_*g*_ are, respectively, within-and between-subject  CVs, derived from biological variation [15].

## 3. Results

 Results are shown in Table 1. Significant differences were observed for the following: RBC, HCT, MCV, PLT, MPV and PDW. No significant differences (*P* > 0.05) were observed by paired Student's *t*-test for: HGB, WBC, LYMPHO, MONO, NEU, EOS, BASO, and LUC also by Wilcoxon ranked-pairs test in MCHC and RDW. Moreover, clinically significant variations, as compared with the current desirable quality specifications [15], were observed only for MPV and PDW.

## 4. Discussion

Our results showed that this new vacuum tubes do not represent a clinical relevant new source of error in clinical laboratory for several routine hematological laboratory parameters. Obviously the most prevalent errors and interferences documented [16–25] as regards the preanalytical phase in laboratory medicine need be prevented, and more so it is important to follow the correct procedures for the collection of diagnostic blood specimens by venipuncture that need be respected and observed by all laboratory quality managers [11, 16–26]. Nevertheless, as regards MPV and PDW our results showed that different brands do represent a novel source of variability. During many years the PDW parameter was not considered important by physicians. Moreover, all physicians know that platelet activation is linked with cardiovascular morbidity [27]. Mean Platelet Volume and Platelet Distribution Width are markers of platelet activation [27–31]. Increased MPV is associated with presence and prognosis of vascular disease, including peripheral, cerebrovascular, and coronary artery disease [32], elevated MPV is found even in diabetes mellitus, especially in the presence of microvascular complications [33–36]. Moreover, PDW can be use to evaluate microvascular complication in diabetes patients [37] or to distinguishing thrombocytopenia in pediatric acute lymphocytic leukemia from immune thrombocytopenia [38]. MPV and PDW are higher in nondiabetic patients with severe obstructive sleep apnoea syndrome and are correlated with different parameters of breathing function during sleep [39]. An accurate investigation of hematological disorders requires appropriate and discretional use of laboratory resources. Therefore, total quality in hematological testing is a prerequisite for clinically reliable results [40]. Modern automated hematology counters can provide clinicians with fast results that are characterized by a high degree of precision and accuracy [41]. Nevertheless, it is known that even little details like fasting time, that is not usually solicited for laboratory hematology complete blood count (CBC), can influence the results interpretation [42]. Different tube brands, possibly with small differences in additives may produce appreciable differences in CBC [43]. As regard our findings, the positive aspect is that different brands do not represent a source of variability for RBC count, total and/or differential WBC count, so such relevant parameters of CBC do not undergo an extra analytical issue that could induce mistakes in diagnoses and/or in the followup of patients [21, 23, 24]. We decided to evaluate these brands of K_3_EDTA because one is among the most frequently used in Europe and in America laboratory settings where the authors are living, and the other has been newly introduced. Obviously, we would like to encourage more quality's laboratory managers from other countries, for example, from China where many other brands of vacuum tubes are employed, to perform a similar evaluation.

## 5. Conclusion

Our results showed that different brand's tubes can represent a clinically relevant source of variations on MPV and PDW. The differences observed among dry EDTA vacuum tubes are probably due to the different preparations, for example, (a) dry EDTA particles of different size and delivery inside the vacuum tube, (b) concentration of dry EDTA, (c) materials of tubes and stoppers. Nevertheless, the above issues can add only little to the possible variations if the final user follows the criteria recommended for item 4.6 of ISO15189 [8], thus giving attention to quality first and foremost, giving secondary importance to the price selling issues. We, considering these K_3_EDTA vacuum tubes validated for routine laboratory hematology tests.

## Figures and Tables

**Table 1:** Variability in hematological parameters from two different brands of K_3_EDTA vacuum tubes.

		Comprehensive results		
Hematological parameters (unit)	Desirable Bias (%)	Tube I	Tube II	Mean % difference
				(*P* value)
RBC^∗^ (×10^6^/*μ*L)	1.7	5.12 ± 0.41 [4.28–5.71]	5.08 ± 0.40 [4.31–5.64]	0.8 (**<0.01**)
HCT^∗^ (%)	1.7	43.5 ± 3.1 [37.9–49.4]	42.9 ± 3.1 [36.9–49.1]	1.4 (**<** **0.01**)
HGB^∗^ (g/dL)	1.8	14.31 ± 1.13 [12.80–16.70]	14.29 ± 1.15 [12.70–16.70]	0.1 (0.56)
MCV^∗∗^ (fL)	1.2	85.9 ± 6.7 [81.4–87.6]	85.6 ± 6.7 [81.1–87.0]	0.4 (**<** **0.01**)
MCHC^∗∗^ (pg)	1.4	28.6 ± 3.1 [26.6–29.3]	28.9 ± 3.2 [26.6–29.3]	−1.0 (0.87)
RDW^∗∗^ (%)	1.7	12.6 ± 1.1 [12.4–13.6]	12.6 ± 1.1 [12.4–13.7]	0.0 (0.94)
WBC^∗^ (×10^3^/*μ*L)	5.6	5.80 ± 1.08 [4.00–7.91]	5.88 ± 1.11 [3.94–8.01]	−1.4 (0.15)
LYMPHO^∗^ (×10^3^/*μ*L)	7.4	1.90 ± 0.66 [0.81–3.33]	1.92 ± 0.70 [0.78–3.64]	−1.0 (0.54)
MONO^∗^ (×10^3^/*μ*L)	13.2	0.35 ± 0.13 [0.17–0.69]	0.36 ± 0.12 [0.21–0.63]	−2.9 (0.27)
NEU^∗^ (×10^3^/*μ*L)	9.0	3.35 ± 0.73 [2.38–4.93]	3.37 ± 0.72 [2.48–5.09]	−0.6 (0.36)
EOS^∗^ (×10^3^/*μ*L)	19.8	0.15 ± 0.10 [0.02–0.36]	0.15 ± 0.10 [0.02–0.37]	0.0 (0.75)
BASO^∗^ (×10^3^/*μ*L)	15.4	0.041 ± 0.016 [0.020–0.070]	0.044 ± 0.020 [0.010–0.090]	−7.3 (0.38)
LUC^∗^ (×10^3^/*μ*L)	NA	0.154 ± 0.048 [0.070–0.260]	0.158 ± 0.054 [0.070–0.270]	−2.6 (0.41)
PLT^∗^ (×10^3^/*μ*L)	5.9	298.3 ± 61.5 [228.0–431.0]	292.7 ± 56.3 [224.0–407.0]	1.9 (**0.02**)
MPV^∗^ (fL)	2.3	8.45 ± 0.69 [7.30–9.80]	8.75 ± 0.84 [7.40–10.6]	**−3.6 (<** **0.01)**
PDW^∗∗^ (%)	1.4	54.4 ± 6.6 [48.9–57.2]	51.8 ± 10.4 [48.8–58.6]	**4.8 (<** **0.01)**

^∗^Normal distribution; the values were mean ± standard deviation (range: minimum-maximum); *P* value represents the significance by paired Student's *t*-test.

^∗∗^Nonnormal distribution; the values were median ± standard deviation (5th–95th percentiles); *P* value represents the significance by Wilcoxon ranked-pairs test.

The bold *P* values are statistically significant (*P* < 0.05), and bold mean % differences represent clinically significant variations, when compared with desirable bias [15].

NA: not available [15].

Tube I: 4.5 mL Labor Import lot 20100505 (concentration of K_3_EDTA inside the tube is not made known by Shandong Weigao Group Medical Polymer, Weihai, China).

Tube II: 1.2 mL S-Monovette lot 0092201 with 1.6 mg K_3_EDTA (Sarstedt, Nümbrecht, Germany).
